# Limited Pre-Speech Auditory Modulation in Individuals Who Stutter: Data and Hypotheses

**DOI:** 10.1044/2019_JSLHR-S-CSMC7-18-0358

**Published:** 2019-08-29

**Authors:** Ludo Max, Ayoub Daliri

**Affiliations:** a Department of Speech and Hearing Sciences, University of Washington, Seattle; b Haskins Laboratories, New Haven, CT; c College of Health Solutions, Arizona State University, Tempe, AZ

## Abstract

**Purpose:**

We review and interpret our recent series of studies investigating motor-to-auditory influences during speech movement planning in fluent speakers and speakers who stutter. In those studies, we recorded auditory evoked potentials in response to probe tones presented immediately prior to speaking or at the equivalent time in no-speaking control conditions. As a measure of pre-speech auditory modulation (PSAM), we calculated changes in auditory evoked potential amplitude in the speaking conditions relative to the no-speaking conditions. Whereas adults who do not stutter consistently showed PSAM, this phenomenon was greatly reduced or absent in adults who stutter. The same between-group difference was observed in conditions where participants expected to hear their prerecorded speech played back without actively producing it, suggesting that the speakers who stutter use inefficient forward modeling processes rather than inefficient motor command generation processes. Compared with fluent participants, adults who stutter showed both less PSAM and less auditory–motor adaptation when producing speech while exposed to formant-shifted auditory feedback. Across individual participants, however, PSAM and auditory–motor adaptation did not correlate in the typically fluent group, and they were negatively correlated in the stuttering group. Interestingly, speaking with a consistent 100-ms delay added to the auditory feedback signal–normalized PSAM in speakers who stutter, and there no longer was a between-group difference in this condition.

**Conclusions:**

Combining our own data with human and animal neurophysiological evidence from other laboratories, we interpret the overall findings as suggesting that (a) speech movement planning modulates auditory processing in a manner that may optimize its tuning characteristics for monitoring feedback during speech production and, (b) in conditions with typical auditory feedback, adults who stutter do not appropriately modulate the auditory system prior to speech onset. Lack of modulation of speakers who stutter may lead to maladaptive feedback-driven movement corrections that manifest themselves as repetitive movements or postural fixations.

Grounded in insights gained from studies on both behavioral and neurological aspects of stuttering, current hypotheses regarding this disorder of speech fluency suggest that the characteristic breakdowns in speech fluency can be attributed to fundamental sensorimotor limitations (Bloodstein, Ratner, & Bernstein-Ratner, 2008; Civier, Bullock, Max, & Guenther, 2013; Max, 2004; Max, Guenther, Gracco, Ghosh, & Wallace, 2004). A primary theme in these contemporary hypotheses is that the neural control of speech movements depends on dynamic interactions between sensory and motor systems, including the prediction of future sensory states based on planned and ongoing motor commands. It is now widely believed that the central nervous system (CNS) controls voluntary movements by making use of internal forward models that are acquired and updated during the process of motor learning (Kawato et al., 2003; Shadmehr, Smith, & Krakauer, 2010; Wolpert, Miall, & Kawato, 1998). Internal forward models of the body's own effector systems and environment are stored neural representations of the mapping between motor commands and their sensory consequences. Conceptual and computational models now generally assume that the CNS predicts sensory consequences of the motor commands using the forward model and an efference copy of the motor commands (Shadmehr et al., 2010; Wolpert, Diedrichsen, & Flanagan, 2011). There is extensive evidence indicating that, when a match is detected between predicted and actual sensory consequences, the CNS modulates its response to the sensory input (Bays, Wolpert, & Flanagan, 2005; Blakemore, Wolpert, & Frith, 1998, 2000; Weiskrantz, Elliott, & Darlington, 1971). For example, electrophysiological studies have shown that cortical responses evoked by self-produced speech sounds (i.e., while speaking) are suppressed in comparison to responses evoked by hearing a played-back version of the same speech sounds, that is, speaking-induced suppression (Chang, Niziolek, Knight, Nagarajan, & Houde, 2013; Houde & Chang, 2015; Houde, Nagarajan, Sekihara, & Merzenich, 2002; Niziolek, Nagarajan, & Houde, 2013). This auditory suppression during speaking is diminished when there is a mismatch between the predicted and actual input, such as when participants hear altered auditory feedback or when the produced speech signals are replaced by those of a different speaker (Chang et al., 2013; Heinks-Maldonado, Nagarajan, & Houde, 2006; Niziolek et al., 2013).

To date, less attention has been paid to a growing body of literature—stemming in large part from animal neurophysiology (Eliades & Wang, 2017; Seki, Perlmutter, & Fetz, 2003) but, recently, also human limb (Voss, Ingram, Haggard, & Wolpert, 2006; Williams & Chapman, 2002) and speech work (Daliri & Max, 2015b; Merrikhi, Ebrahimpour, & Daliri, 2018; Mock, Foundas, & Golob, 2015)—indicating a wholly separate, more direct influence of sensory predictions during movement planning on task-relevant sensory systems. Such a direct influence is reflected in findings demonstrating that (a) the response to externally generated inputs is also modulated during movement (Altenmüller, Berger, Prokop, Trippel, & Dietz, 1995; Chapman, Jiang, & Lamarre, 1988; Eliades & Wang, 2002; Numminen, Salmelin, & Hari, 1999; Sowman, Brinkworth, & Türker, 2010), (b) sensory modulation occurs at levels as low as the spinal cord (Chapman et al., 1988; Seki et al., 2003), and (c) sensory modulation can already start hundreds of milliseconds before movement onset or when movement is prevented altogether (Cohen & Starr, 1985; Voss et al., 2006; Walsh & Haggard, 2008, 2010; Williams & Chapman, 2002).

Given that premovement sensory modulation mechanisms had remained almost entirely unexplored in speech production, we initiated a series of experiments to study motor-to-sensory influences during speech movement planning in both typical speakers and individuals who stutter. More specifically, we developed and fine-tuned a new experimental paradigm (first described in Max, Daniels, Curet, & Cronin, 2008) in which auditory evoked potentials (AEPs) are recorded in response to probe tones that are played either immediately prior to speaking (i.e., during speech planning in a delayed-response speaking task) or at the equivalent time in no-speaking control conditions (i.e., silent reading or silently viewing nonlinguistic symbols). All conditions are completed in separate blocks of trials; thus, participants always know ahead of time whether or not they should speak in a given trial. All conditions also include trials without probe tone so that a separate evoked potential associated with time-locked nonauditory processes (e.g., visual responses, word recognition, word reading) can be subtracted from the AEP in order to obtain the best possible estimate of the auditory response itself. As a measure of pre-speech auditory modulation (PSAM), we then calculate the change in amplitude of the long-latency AEP components N1 and P2 during the planning phase prior to speech initiation relative to the no-speaking control condition.

Our results from several studies convincingly showed that the CNS already modulates the auditory system during speech planning prior to movement onset. These PSAM results were especially intriguing in the context of theoretical models of stuttering: Based on our perspective formulated within the broader theoretical context of control systems theory (Max, 2004; Max et al., 2004), individuals who stutter may have difficulties with the use of sensory predictions to successfully prepare task-relevant sensory systems for their subsequent role in feedback monitoring. In the following sections, we briefly describe our studies that have addressed questions regarding PSAM in both adults who stutter and adults who do not stutter.

## Do Adults Who Stutter Show Atypical PSAM?

**Figure 1.**
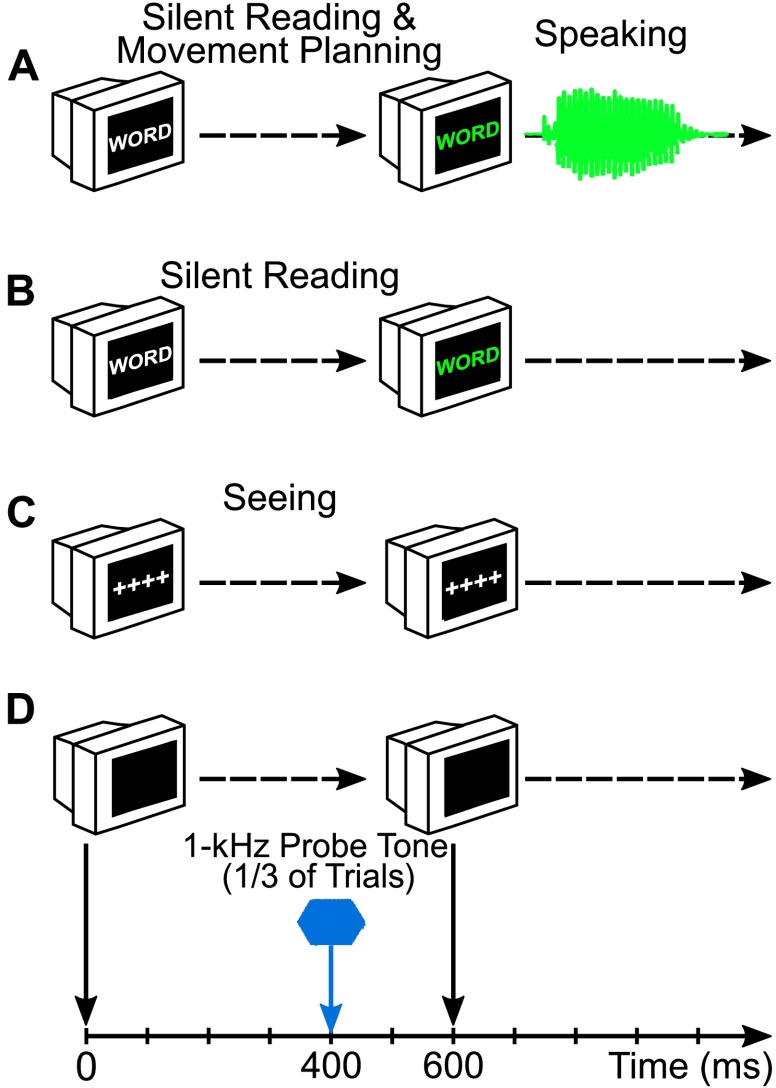
Schematic of the experimental paradigm. The experiment included three conditions: (A) speaking, (B) silent reading, and (C) seeing. (D) An auditory stimulus (1 kHz, 50-ms duration, 75 dB SPL) was presented 400 ms after initial appearance of the stimulus word (in the speaking and silent reading conditions) or symbols (in the seeing condition) in one third of the trials of each condition, whereas no auditory stimulus was presented in the remaining trials. Adapted from Daliri and Max (2015b): *Brain and Language,* Vol. 143, A. Daliri and L. Max, “Modulation of Auditory Processing During Speech Movement Planning is Limited in Adults Who Stutter,” pp. 59–68, Copyright © 2015, with permission from Elsevier.

The primary goal of our first PSAM study with both participants who stutter and participants who do not stutter (Daliri & Max, 2015b) was to investigate whether stuttering is associated with abnormalities in pre-speech sensory modulation in the auditory system. To address this question, we used our previously developed paradigm (schematically illustrated in Figure 1) with a delayed-response speaking task and no-speaking control conditions (Max et al., 2008). We used AEP analyses to examine the modulation of auditory cortical activity in response to probe stimuli presented through insert earphones during speech movement planning and in control conditions without movement planning. We tested adults who stutter (*n* = 12) and adults who do not stutter (*n* = 12) who were native speakers of American English and right-handed. They reported no history of psychological, neurological, or communication disorders (other than stuttering in the stuttering group), and all had normal binaural hearing thresholds (≤ 20 dB HL at all octave frequencies of 250–4000 Hz).

The participants completed nine blocks of trials (three blocks for each of three conditions), with each block including only speaking trials or no-speaking trials. Thus, even though the order of blocks was counterbalanced, participants were aware at all times whether or not the task involved producing speech. In each trial for the speaking condition, a monosyllabic word (three to five letters long) appeared in white characters on a computer monitor. After 600 ms, the color of the word changed to green as a “go” signal for the participant to produce the word overtly. The same procedure with the same sequence of visual events occurred in a silent reading condition, except that participants were instructed to not produce the words aloud. Thus, the silent reading condition served as a direct control condition for the speaking condition as it was specifically designed to eliminate speech movement planning. A seeing condition served as a further control condition by also eliminating cognitive–linguistic processes associated with reading, word recognition, and so forth. This seeing condition included the same sequence of events as in the silent reading condition, except that nonlinguistic symbols (“++++”) rather than monosyllabic words were shown on the computer monitor.

To investigate adjustments in auditory processing during speech movement planning, a probe stimulus (1 kHz, 75 dB SPL, 50-ms duration, 10-ms rise and fall times) was presented during one third of the 270 trials per condition (further referred to as *tone trials,* with the remaining trials referred to as *no-tone trials*). The probe stimulus was delivered 400 ms after presentation onset of the word or symbols in white characters (thus, 200 ms prior to the change from white characters to green characters). Pilot testing with auditory stimuli presented at various time points before the color change (see Max et al., 2008) revealed that this time point 400 ms after initial presentation of the visual stimulus offered the best balance between avoiding visually evoked potentials and movement-related artifacts.

We recorded high-density electroencephalography (EEG) data from 128 electrode sites on the scalp (according to an extension of the 10–10 electrode system) along with electrooculograms, orofacial electromyograms, and speech signals. Data preprocessing included rereferencing (relative to offline reconstructed average mastoids), filtering (low-pass filter with a cutoff frequency of 50 Hz), and epoching (−100 to 400 ms relative to probe stimulus or equivalent time in no-tone trials). Each epoch was baseline corrected using the mean amplitude of the prestimulus period. Outlier epochs containing EEG amplitudes greater than ± 70 μV, eye movements, blinking, or muscle artifacts were excluded. Artifact-free epochs were then averaged to derive evoked potentials for each condition's tone trials and no-tone trials separately. Evoked potentials for the tone trials reflect auditory and nonauditory processing (e.g., motor, linguistic, cognitive, and visual processes), whereas evoked potentials for the no-tone trials reflect only the nonauditory processes. Thus, for each participant in each condition, we subtracted the evoked potential for no-tone trials from the evoked potential for tone trials in order to remove the influence of nonauditory processes (note that the validity of this approach is confirmed by achieving standard AEP morphology after the subtraction).

Amplitudes and latencies of the N1 component (the largest negative peak between 70 and 130 ms after stimulus onset) and the P2 component (the largest positive peak between 150 and 250 ms after stimulus onset) in the final AEP were extracted from a subset of EEG electrodes over central and lateral regions of the scalp. Replicating a result also reported in Max et al. (2008), the absence of any AEP difference between the entirely nonlinguistic seeing task and the silent reading task confirmed that the latter can serve as an appropriate reference for defining PSAM. Thus, we quantified individual participant PSAM as the difference in N1 or P2 amplitude in the silent reading condition versus the speaking condition. Doing so on the basis of a within-subject comparison is based on fundamental considerations related to electrophysiological recordings. Between-subjects comparisons of EEG data would reflect not only neurophysiological differences between the groups but also anatomical intersubject variation in the effects of skin and bone (including the thickness of different layers) on the extracranially recorded electrical fields (Nunez & Srinivasan, 2006). For this reason, within-subject comparisons and Group × Condition interactions are more meaningful and generally recommended (Luck, 2014).

**Figure 2.**
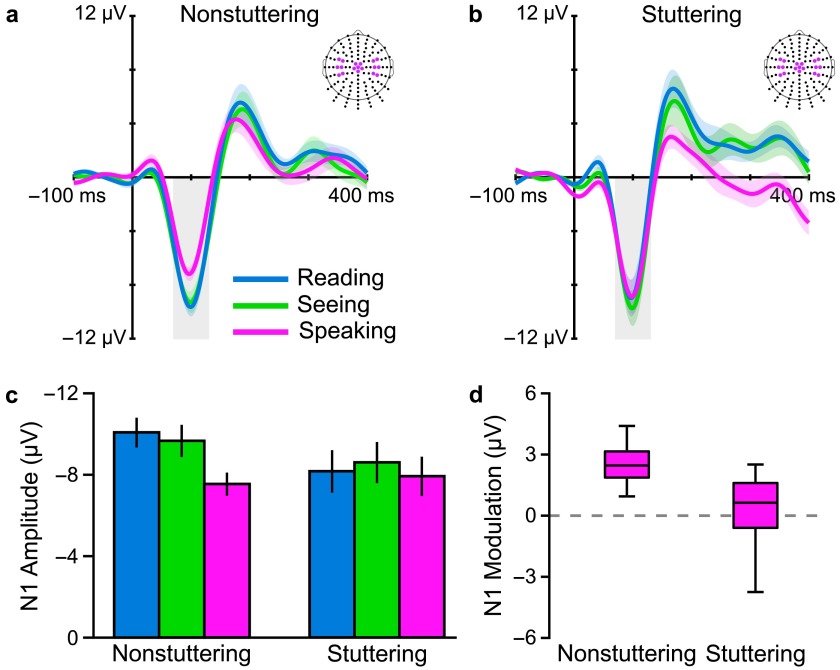
Grand-averaged auditory evoked potentials (across participants and across electrodes in three regions of interest marked on the scalp maps) for (a) adults who do not stutter and (b) adults who stutter. The two groups differ in modulation of the N1 component (gray box) in the speaking condition relative to the silent reading and seeing control conditions. (c) Group-averaged amplitudes (with standard errors of the mean) of the N1 component in each condition illustrate the Group × Condition interaction. N1 amplitude of the nonstuttering group was smaller in the speaking condition relative to both control conditions; N1 amplitude of the stuttering group was similar across all three conditions. (d) A boxplot illustrates the consistency of N1 modulation (N1 modulation = |N1_Reading_| − |N1_Speaking_|; thus, positive values indicate an amplitude reduction in the speaking condition) among speakers who do not stutter as compared with the absence of this phenomenon among speakers who stutter. Adapted from Daliri and Max (2015b) *Brain and Language,* Vol. 143, A. Daliri and L. Max, “Modulation of Auditory Processing During Speech Movement Planning is Limited in Adults Who Stutter,” pp. 59–68, Copyright © 2015, with permission from Elsevier.

The main results from this study all related to N1 amplitude and are shown in Figure 2 (results for P2 amplitude or P1 and P2 latencies showed no statistically significant Group × Condition interactions). Panels a and b display AEP waveforms for the stuttering and nonstuttering groups in the speaking, silent reading, and seeing conditions; Panel c summarizes the extracted amplitude values for N1 in a bar graph; and Panel d contains a boxplot depicting each group's amount of PSAM. Data obtained from the typically fluent adults confirmed our hypothesis that, consistent with a predictive modulation of auditory processing before speech sound production, amplitude of the auditory N1 (in Figures 2a and 2b highlighted with a gray-shaded area) was statistically significantly reduced before speaking as compared with both the silent reading and seeing conditions (see Figure 2c). Data from the adults who stutter, on the other hand, showed no difference in N1 amplitude between the speaking condition and either of the nonspeaking conditions (see Figures 2a– 2c). Consequently, the stuttering group's modulation of N1 amplitude (defined as N1 modulation = |N1_Reading_| − |N1_Speaking_|) during speech planning was substantially smaller than that observed in typically fluent adults (see Figure 2d). Thus, overall, our study demonstrated that adults who stutter show atypical, or even entirely absent, PSAM.

## Does the Lack of PSAM in Adults Who Stutter Reflect a General Auditory Prediction Deficit?

Findings from the first study suggested that adults who stutter are less efficient in using auditory prediction during speech movement planning. However, the study did not address whether the observed absence of PSAM in speakers who stutter was due to an inefficient planning of motor commands (which presumably have a modulating influence) or due to inefficiencies in the forward modeling process itself. Thus, in a follow-up study (Daliri & Max, 2015a), we investigated whether adults who stutter also differ from adults who do not stutter when the forward modeling of auditory input is not based on the simultaneous preparation of motor commands.

To address this question, we tested 10 adults who stutter and 10 adults who do not stutter (participant inclusion/exclusion criteria were as described above) after adding a new condition to the previous paradigm. We recorded AEPs in response to probe auditory stimuli presented through insert earphones in the following three conditions (each condition consisting of three blocks of 90 trials): during movement planning prior to speaking, prior to listening to one's own speech playback, and during silent reading. Each block contained only trials from one single condition, and participants were instructed prior to the beginning of the block whether they should speak, listen, or read silently on each trial. The speaking condition was identical to that used in the prior study; that is, auditory prediction was based on speech movement planning. In the listening condition, a monosyllabic word also appeared in white characters on a black background, and after 600 ms, the color of the word also changed to green. However, participants did not read the words out aloud. Instead, after the word appeared on the screen, we played back a recording of the participant's own production of that same word at the same intensity and with the same onset latency as produced by the participant in the speaking condition (one block of trials for the speaking condition was always completed first in the total set of nine blocks such that a recording of each word was available). Thus, in the listening condition, auditory prediction was based on the expectation of hearing one's own production of the displayed word, but it occurred in the absence of speech movement planning. Surface electromyographic signals from several orofacial muscles were inspected to exclude any trials with noticeable muscle activity, but we did not notice a trend for undesirable muscle activity to occur during these passive listening trials. As the third condition, silent reading again served as a control activity that did not involve any type of auditory prediction. To measure auditory modulation, we delivered a probe tone (1 kHz, 75 dB SPL, 40 ms) at 400 ms after presentation of the word in 40% of the trials in each condition. Processing and analysis steps (including the subtraction of an evoked potential recorded in no-tone trials from the AEP recorded in tone trials) were as described above for the first study.

**Figure 3.**
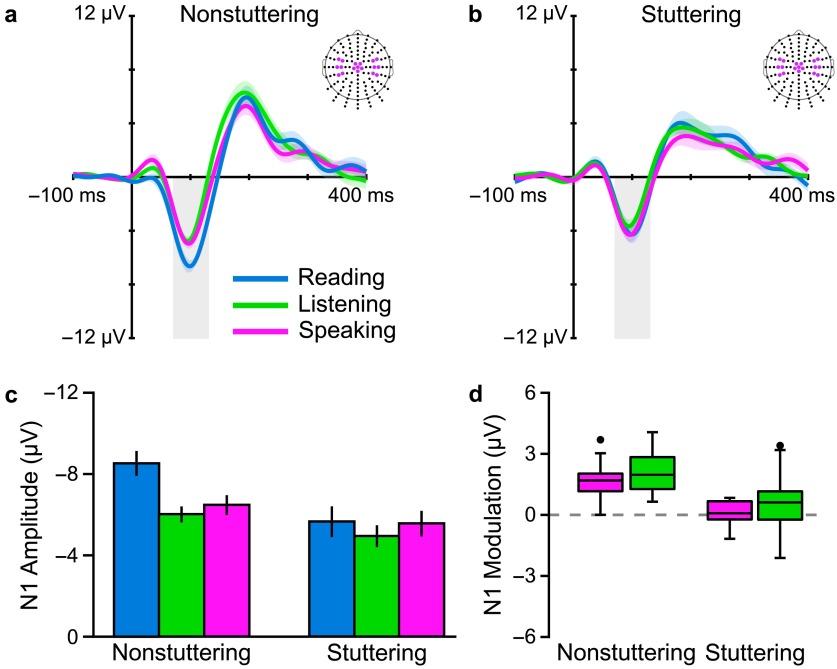
Grand-averaged auditory evoked potentials (averaged over the colored electrodes in the scalp maps) for (a) adults who do not stutter and (b) adults who stutter. We found that N1 amplitude of the participants who do not stutter in both the speaking and listening conditions was smaller than that in the silent reading condition; however, (c) N1 amplitude of the participants who stutter was similar in all three conditions (error bars indicate standard errors of the mean). (d) The boxplot illustrates participant distribution in terms of N1 modulation in the speaking and listening conditions for the two groups (i.e., modulation calculated such that positive values indicate an amplitude reduction relative to the silent reading condition). Adapted from Daliri and Max (2015a), *Brain and Language,* Vol. 150, A. Daliri and L. Max, “Electrophysiological Evidence for a General Auditory Prediction Deficit in Adults Who Stutter,” pp. 33–44, Copyright © 2015, with permission from Elsevier.

A first important finding was that we once again replicated our previous result that adults who do not stutter clearly modulate their auditory system during speech planning prior to movement onset (i.e., they show a reduction in N1 amplitude in the speaking condition relative to the silent reading condition). Second, we also replicated that this mechanism is lacking in adults who stutter. Third, as a novel finding, adults who do not stutter also showed a similar auditory modulation when expecting to hear a played-back version of their own speech (i.e., there is a reduction in N1 amplitude in the listening condition relative to the silent reading condition), and this modulation too was very limited in adults who stutter. Overall, the results—summarized in Figure 3—provided direct electrophysiological evidence for a generalized auditory prediction deficit in adults who stutter. In particular, the findings suggest that the lack of PSAM in adults who stutter is likely not a result of inefficient or incorrect motor command planning but of inefficiencies in the forward modeling of future auditory inputs, regardless of whether the prediction is based on self-generated motor commands or other information.

## Determining the Functional Relevance of PSAM: Is the Effect Sound Specific?

When modulation occurs in a sensory response to movement itself—such as reduced auditory cortex responses in magnetoencephalographic recordings during self-generated versus played-back speech—the data are typically interpreted as reflecting a partial suppression of self-generated inputs (Heinks-Maldonado, Mathalon, Gray, & Ford, 2005; Heinks-Maldonado et al., 2006; Houde et al., 2002). This phenomenon is considered critical for the ability to differentiate between the consequences of one's own actions and stimuli created by external sources (Blakemore, Wolpert, & Frith, 1999; Blakemore et al., 2000). In the context of the theoretical framework discussed in the introduction, such sensory suppression operates at a level where afferent inputs have already been sufficiently processed in the primary sensory cortex to allow a comparison with an earlier prediction signal, and after this comparison has been made, the response can be partially suppressed if a match is indeed detected. It is not at all clear, however, whether or not the PSAM phenomenon before movement onset also reflects an actual suppression of afferent input. In robotics, for example, it would be ill-advised to suppress in a global manner the incoming signals from contact and force sensors that feed back information about an actuator's movements. It is therefore helpful to be reminded of the fact that a reduction in the amplitude of a given evoked potential component (i.e., the scalp-recorded sum of all electrical potentials from numerous intracranial dipole sources) does not necessarily indicate an actual suppression of the specific cognitive process of interest. The notion that the CNS would start applying a general suppression of auditory pathways during the planning phase prior to speech onset is also difficult to reconcile with studies demonstrating that auditory feedback is the primary modality driving speech motor learning (e.g., Feng, Gracco, & Max, 2011).

Of course, this raises the question of what the functional relevance of the PSAM phenomenon might be. Based on current theoretical models of speech motor control, when the CNS plans to produce a specific speech sound or sequence of speech sounds, it accurately predicts the auditory consequences of the planned movements, and it uses this prediction to inform the auditory cortex about expected feedback input (Chang et al., 2013; Houde & Nagarajan, 2011; Niziolek et al., 2013; Tourville & Guenther, 2011). If an initial priming of sensory (in this case auditory) systems does not serve to suppress afferent inputs, then one might hypothesize that it does, in fact, play a role in engaging or even enhancing processes involved in sensory feedback monitoring. With the aim to start unraveling the functional relevance of PSAM, we conducted a separate study with only individuals who do not stutter (*n* = 9) in which we investigated whether PSAM varies depending on the nature of the auditory stimulus that is used to probe auditory processing immediately prior to speech onset (Daliri & Max, 2016). The study included a speaking condition and a silent reading condition identical to those used in our previous studies. However, rather than using only one type of auditory stimulus to probe auditory processing, this time, AEPs were elicited by either pure tones or truncated speech syllables. Both stimuli were 40 ms in duration and presented at 75 dB SPL through insert earphones. They were again presented 200 ms before the “go” signal during the instructed delay period in the speech condition or at the equivalent time point in the silent reading control condition (i.e., 400 ms after appearance of the word). Also, as in the procedures described above, nonauditory contributions to the AEP were removed by subtracting the evoked potential recorded during no-tone trials during which no probe stimulus was presented but the task was otherwise identical.

**Figure 4.**
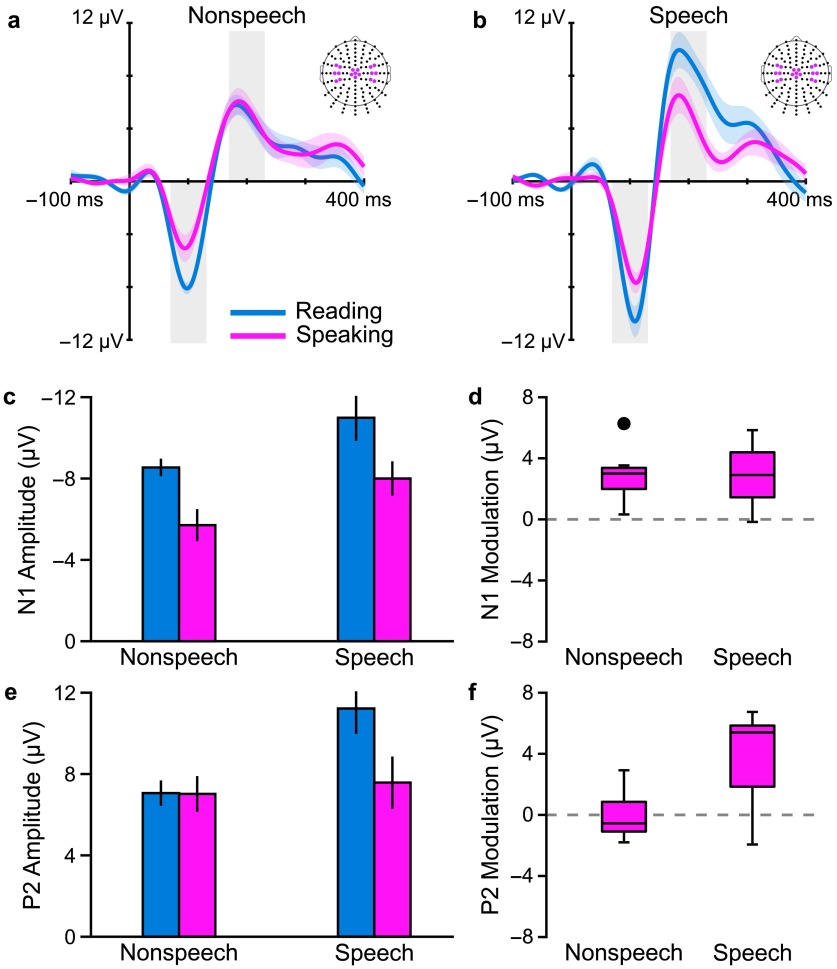
Nonstuttering speakers' grand-averaged auditory evoked potentials in response to (a) nonspeech stimuli (1-kHz tone) and (b) speech stimuli (/da/ syllable) that were presented either prior to vocalization in a speaking condition (pink) or at the same time point in a silent reading condition (blue). We examined changes in amplitude of the N1 and P2 components (highlighted by the gray-shaded areas in a and b) in the speaking condition versus the reading condition to determine auditory modulation for each type of stimulus. N1 modulation occurred to a similar extent in responses to speech and nonspeech auditory stimuli; (c) N1 amplitudes in the speaking and reading conditions; error bars indicate standard errors of the mean and (d) Boxplot of auditory modulation calculated as |N1_Reading_| − |N1_Speaking_|. P2 modulation occurred only in responses elicited by speech stimuli: (e) P2 amplitudes and (f) P2 modulation. All data averaged across the colored electrodes in three regions of interest. Adapted from Daliri and Max (2016), Creative Commons Attribution License (CC BY), Copyright © 2016 Daliri and Max.

The obtained results showed no statistically significant effect of stimulus type on modulation of the N1 component that reflects early stages of auditory processing approximately 100 ms after stimulus onset. For this N1 component, PSAM was observed regardless of the speech versus nonspeech nature of the probe stimulus. However, the P2 component that reflects later stages of auditory processing approximately 200 ms after stimulus onset showed significant PSAM only for the speech stimulus (see Figure 4). Thus, the results indicate that PSAM during movement planning has a global effect on early auditory processing stages but a speech-specific effect on later auditory processing stages. In other words, at least in terms of the amplitudes of scalp-recorded AEP components, preparing to speak aloud affects the initial cortical processing of auditory input in a global manner but subsequent higher level cortical processing in a speech-specific manner. As a tentative working hypothesis, we propose that this finding is consistent with a conceptual model in which different phases of auditory modulation during speech planning may reflect ongoing neural processes involved in priming and selectively biasing the auditory system for its role in monitoring auditory feedback during speech production (for more details, see the General Discussion section below).

## Determining the Functional Relevance of PSAM: Does the Effect Relate to Auditory–Motor Learning?

As a next step toward investigating both the functional relevance of PSAM itself and the potential role of a lack of PSAM in stuttered speech disfluencies, we speculated that, if the phenomenon reflects processes involved in priming the auditory cortex for the subsequent monitoring of incoming feedback, then individuals with limited PSAM may also show limited auditory–motor learning when speech is produced with experimentally altered auditory feedback. This rationale is based on the fact that such auditory–motor learning is critically dependent on sensory prediction errors (i.e., discrepancies between predicted and actual feedback) and that the detection of these prediction errors requires an accurate monitoring of the actual feedback signal. In other words, we hypothesized a functional role for PSAM in optimizing feedback monitoring without implying that PSAM and auditory–motor learning share the same neural mechanisms.

For this purpose, we designed a new study (Daliri & Max, 2018) with both adults who stutter (*n* = 13) and adults who do not stutter (*n* = 13) in which we examined the relationship between PSAM and adjustments in speech articulation based on experimental manipulations of auditory feedback. Participant inclusion/exclusion criteria were as in the prior studies. In one session, participants completed a sensorimotor adaptation paradigm that allowed us to quantify, on an individual basis, the extent of speech motor learning induced by altered auditory feedback. While producing monosyllabic consonant–vowel–consonant (CVC) words, participants heard auditory feedback that was digitally altered in real time during a perturbation phase but unaltered during a preceding baseline phase and a following postperturbation phase. Specifically, a professional-quality vocal processor (VoiceOne, TC Helicon) shifted all formant frequencies in the feedback signal 250 cents up (1 octave = 1200 cents) during all trials in the perturbation phase. Offline, we measured each participant's produced first (F1) and second (F2) formant frequencies in the baseline, perturbation, and postperturbation phases. Each individual's extent of auditory–motor learning was quantified as the change in both F1 and F2 at the end of the perturbation phase relative to baseline and by an overall adaptation index that combined the change in both formants. In a second session, the same participants completed our PSAM paradigm already described above. Pure tones were used as the probe stimuli presented during speech movement planning or at the equivalent point in time during silent reading. Processing of the EEG data and analysis of the AEPs were as described above. The experiment also included an extra speaking condition with delayed auditory feedback (DAF), but that condition served to address a separate research question (see the Is Stuttering Individuals' Lack of PSAM “Reversible”? section below). Thus, all PSAM results summarized in this section relate to the speaking condition with typical auditory feedback.

**Figure 5.**
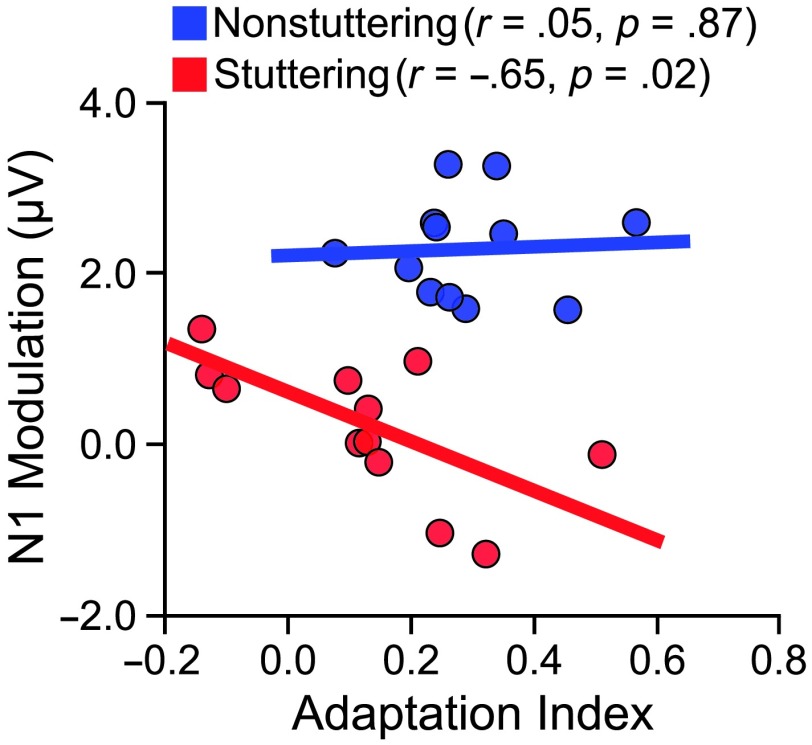
Relation between pre-speech modulation of the auditory evoked potential N1 component and speech adaptation to altered auditory feedback. Participants completed an auditory–motor adaptation paradigm that allowed us to quantify each participant's adaptation to formant-shifted auditory feedback. The same participants also completed our standard pre-speech auditory modulation (PSAM) paradigm that allowed us to quantify each participant's extent of N1 and P2 modulation prior to speech onset. N1 PSAM was not statistically significantly correlated with the amount of adaptation for speakers who do not stutter (*r* = .05, *p* = .87), and it was negatively correlated with the amount of adaptation for speakers who stutter (*r* = −.65, *p* = .02). Neither group showed a significant correlation between adaptation and P2 PSAM (*p* > .349). Adapted from Daliri and Max (2018): *Cortex*, Vol. 99, A. Daliri and L. Max, “Stuttering Adults’ Lack of Pre-Speech Auditory Modulation Normalizes When Speaking With Delayed Auditory Feedback,” pp. 55–68, Copyright © 2018, with permission from Elsevier.

Results from the sensorimotor adaptation task showed statistically significant F1 and F2 adaptation only in the nonstuttering group and not in the stuttering group. Similarly, results from the PSAM paradigm showed statistically significant N1 modulation (speaking vs. silent reading) only in the nonstuttering group and not in the stuttering group. Most important for this section's research question, we asked whether there is a relation between auditory–motor adaptation in the first session and PSAM in the second session. Thus, we calculated the Pearson correlation between the overall index of adaptation to formant-shifted auditory feedback and the extent of auditory N1 modulation prior to speaking (see Figure 5). Contrary to our hypothesis, PSAM was not statistically significantly correlated with the amount of adaptation for speakers who do not stutter (*r* = .05, *p* = .87), and it was negatively correlated with the amount of adaptation for speakers who stutter (*r* = −.65, *p* = .02). In other words, participants who stutter and showed more PSAM showed less auditory–motor adaptation. Neither group showed a significant correlation between adaptation and auditory P2 modulation prior to speaking.

In summary, our prior finding of limited N1 PSAM in adults who stutter was again replicated. In addition, we confirmed that the same adults who stutter also showed limitations in auditory–motor speech learning. Thus, based on the group-level comparisons, these data could be considered in agreement with the hypothesis that PSAM enhances (rather than suppresses) feedback monitoring. However, this relationship between the two variables did not hold up within each group: At the participant level, the two variables were not correlated for speakers who do not stutter and negatively correlated for speakers who stutter. The stuttering group's negative correlation between PSAM and auditory–motor adaptation is difficult to interpret in light of the fact that, as mentioned, speakers who do not stutter showed a greater extent of PSAM, yet they also showed a greater, rather than smaller, extent of adaptation (indicating that more PSAM by itself is not associated with less adaptation). Nevertheless, the overall pattern of results suggests that limited PSAM is not directly related to limited auditory–motor adaptation at the level of individual speakers. New studies are underway in our laboratory to further explore the relationship, if any, between PSAM and auditory feedback monitoring.

## Is Stuttering Individuals' Lack of PSAM “Reversible”?

Another hypothesis derived from the notion that PSAM may reflect neural changes associated with preparing the auditory system for feedback monitoring is that exposure to nonuseful auditory feedback would lead to changes in the PSAM phenomenon. We speculated that adding a consistent and noticeable delay to the auditory feedback signal would reduce speakers' ability to rely on this channel for online monitoring when producing short, monosyllabic words. We also speculated that this reduced ability to rely on auditory feedback, in turn, would lead to reduced activation of the mechanisms responsible for PSAM. We therefore included in the study discussed in the Determining the Functional Relevance of PSAM: Does the Effect Relate to Auditory–Motor Learning? section not only the described speaking condition with typical, nonaltered auditory feedback (NAF) but also a speaking condition during which DAF was predictably applied on each trial.

**Figure 6.**
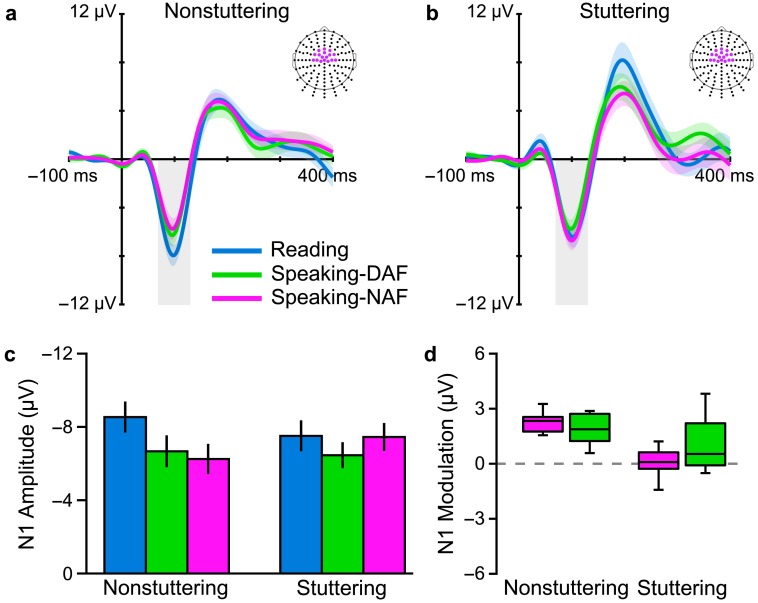
Grand-averaged auditory evoked potentials of (a) nonstuttering and (b) stuttering groups in response to probe tones presented during speech planning in a condition with nonaltered auditory feedback (NAF), during speech planning in a condition with delayed auditory feedback (DAF), and at the equivalent time points in a condition with only silent reading. Group-averaged N1 amplitudes in each of the conditions are shown in (c) (error bars correspond to standard errors of the mean). (d) Pre-speech modulation of the N1 component (|N1_Reading_| − |N1_Speaking_|) for the nonstuttering group decreased in the DAF condition relative to the NAF condition, whereas for the stuttering group, it increased for the same comparison. As a result, the between-groups difference in pre-speech modulation during speaking with NAF disappeared when speaking with DAF. Adapted from Daliri and Max (2018): *Cortex*, Vol. 99, A. Daliri and L. Max, “Stuttering Adults’ Lack of Pre-Speech Auditory Modulation Normalizes When Speaking With Delayed Auditory Feedback,” pp. 55–68, Copyright © 2018, with permission from Elsevier.

As has been the case in all our PSAM studies to date, participants produced short CVC words after a “go” signal that was presented 600 ms after initial presentation of the word on a computer monitor. For each production, however, the auditory feedback signal presented through insert earphones was experimentally delayed by 100 ms. Pure-tone stimuli (1 kHz, 40 ms, 10-ms rise and fall times) were presented through the insert earphones during the speech planning phase prior to movement onset (400 ms after initial visual presentation of the target word). After again removing nonauditory contributions to the AEP by means of the no-tone trial subtraction procedures described above, the AEP responses to these pure-tone stimuli during speech planning in the DAF condition were compared to the AEP responses obtained at the equivalent time point in the silent reading condition. The amount of PSAM for this DAF condition (change in N1 amplitude in the DAF speaking condition vs. the silent reading control condition) was then compared with the amount of PSAM in the NAF condition discussed above in the Determining the Functional Relevance of PSAM: Does the Effect Relate to Auditory–Motor Learning? section (see Figure 6).

Overall, we found that the effect on PSAM of speaking with DAF differed between the stuttering and nonstuttering groups. For eight of the 12 participants who do not stutter, less PSAM was observed in the DAF condition as compared with the NAF condition. Interestingly, however, nine of the 12 participants who stutter showed an increase, rather than decrease, in PSAM in the DAF condition versus the NAF condition. In fact, as a group, the individuals who stutter showed a statistically significant modulation of the auditory N1 response only before speaking with DAF and not before speaking with NAF. This increase in PSAM for the speakers who stutter was sufficiently large for the between-group difference that we had consistently observed in all our studies to be statistically nonexistent in this DAF condition.

For the group of participants who stutter, we also determined the correlation between the amount of PSAM in either the NAF condition (from the Determining the Functional Relevance of PSAM: Does the Effect Relate to Auditory-Motor Learning? section above) or the DAF condition (this section) and stuttering frequency data from a preceding clinical assessment. When speaking with NAF, there was no statistically significant correlation between either of the two PSAM values and stuttering frequency. When speaking with DAF, however, PSAM in the DAF condition was statistically significantly correlated with stuttering frequency (*r* = .66, *p* = .02). In other words, when hearing auditory feedback with a 100-ms delay, those participants with a higher frequency of stuttering moments showed the most PSAM.

Thus, the replicated finding of a lack of PSAM in adults who stutter (Daliri & Max, 2015a, 2015b, 2018) applies only to speaking with typical NAF. PSAM mechanisms are activated in this population when the task involves speaking with DAF. It should be noted that, although the probe auditory stimulus is always presented prior to producing the word for each individual trial (and thus prior to hearing the delayed feedback for that trial), participants know after the first few trials in the DAF condition that they will always hear their own speech with a delay. Thus, it appears to be a general awareness or expectation of DAF being “on” in this condition that leads to the activation of the processes responsible for PSAM of the individuals who stutter. We believe that this is a promising experimental finding in the context of a large literature on the fluency-enhancing effects of DAF for many individuals who stutter (for a review, see Bloodstein et al., 2008). Whether the DAF-based “normalization” of PSAM of speakers who stutter as demonstrated in Daliri and Max (2018) and such DAF-based fluency enhancements are directly or indirectly related remains to be determined in future studies.

## General Discussion

Numerous previous publications have suggested that stuttering is associated with deficiencies in auditory feedback monitoring or auditory–motor integration (Cai, Beal, Ghosh, Guenther, & Perkell, 2014; Cai et al., 2012; Civier, Tasko, & Guenther, 2010; Daliri, Wieland, Cai, Guenther, & Chang, 2017) or sensorimotor integration in general (Daliri, Prokopenko, Flanagan, & Max, 2014; Daliri, Prokopenko, & Max, 2013; Max, 2004; Namasivayam, van Lieshout, McIlroy, & De Nil, 2009). However, it remains unknown which specific aspects of sensorimotor integration are deficient in individuals who stutter. Over the past two decades, the neural control of movement literature has made it increasingly clear that sensorimotor integration involves, among other aspects, substantial involvement of neural processes related to predicting the sensory outcomes of planned motor commands (Shadmehr et al., 2010; Wolpert & Flanagan, 2001; Yang, Wolpert, & Lengyel, 2016). Such predictions are used both to optimize motor commands and to prepare task-relevant sensory systems for their subsequent role in processing incoming feedback signals during movement execution (Wolpert et al., 2011; Wolpert & Flanagan, 2001). Given the many suggestions that auditory–motor integration deficiencies play a role in stuttering, the program of research reviewed here has investigated this latter role of sensory prediction by investigating stuttering and nonstuttering speakers' modulation of the auditory system even before speech movement onset.

The results from this programmatic series of studies can be summarized as follows. First, adults who do not stutter show a clear modulation of auditory processing during speech planning prior to movement onset—a finding that we have replicated multiple times (Daliri & Max, 2015a, 2015b, 2016, 2018; Max et al., 2008). We refer to this phenomenon as PSAM. Second, adults who stutter show very limited, if any, PSAM, at least when speaking with typical, unaltered auditory feedback—another finding that we have replicated after the original report (Daliri & Max, 2015a, 2015b, 2018). Third, when participants know that they will hear their own prerecorded speech played back to them rather than actively producing it, a similar between-group difference is observed with PSAM occurring in the speakers who do not stutter but not in the speakers who stutter (Daliri & Max, 2015a). Fourth, PSAM affects the auditory system's response to probe stimuli regardless of whether those stimuli are pure tones or truncated speech syllables, but only the response to speech stimuli shows a modulation also during later processing stages (affecting P2 ~200 ms after probe stimulus onset), in addition to the general modulation that occurs during earlier processing stages (affecting N1 ~100 ms after probe stimulus onset; Daliri & Max, 2016). Fifth, compared with typically fluent control participants, adults who stutter show both less PSAM when speaking with unaltered auditory feedback and less auditory–motor adaptation in a separate speaking task with formant-shifted auditory feedback; it remains unknown, however, why—at the individual participant level—these two measures are not correlated for typically fluent speakers whereas they are negatively correlated for speakers who stutter (Daliri & Max, 2018). Sixth, when speaking in a condition in which a consistent 100-ms delay is added to the auditory feedback signal, PSAM is reduced in most fluent speakers and increased in most speakers who stutter; in fact, with such a delay present, there is no longer a difference in PSAM between the two participant groups (Daliri & Max, 2018).

Thus, taken together, the evidence is compelling in indicating that the sensorimotor system typically uses predictive mechanisms to modulate auditory processing during speech planning prior to movement onset, that these mechanisms are not operating in the same manner in adults who stutter, and that this lack of PSAM in adults who stutter is eliminated when speaking with delayed feedback. What remains unclear is (a) what exactly the functional relevance is of the PSAM phenomenon and (b) whether less active PSAM mechanisms do play a role in stuttered speech disfluencies. In our studies, to date, participants have produced only short, monosyllabic CVC words, and as a result, participants who stutter have been able to produce almost all utterances fluently (note that, even for a single participant, many disfluent productions would be necessary to derive a separate AEP for probe stimuli preceding such disfluent productions and, therefore, no such analyses have been completed).

As a current working hypothesis, we speculate that stuttering individuals' limited use of auditory prediction to prime the auditory system prior to movement onset may result in an inefficient use of auditory feedback during speech production. Given that we found no relation between PSAM and auditory–motor learning for speakers who do not stutter, we speculate that the affected monitoring mechanisms may play a role primarily in online feedback-driven corrections rather than in corrections over a longer timescale such as those driving auditory–motor learning—an experiment directly testing this hypothesis is currently ongoing in the first author's laboratory (see also Eliades & Tsunada, 2018). When producing individual monosyllabic words, feedforward mechanisms may suffice for fluent movement execution (Kim & Max, 2014). For the production of complex multisyllabic words and sequences of syllables combined into complete utterances, however, feedback monitoring and feedback-driven corrections may play a role of greater importance. During such longer speech sequences, not appropriately modulating the auditory system for its role in feedback monitoring may lead to unnecessary and disruptive attempts at correcting ongoing movements, and such repairs may contribute to the fluency breakdowns that form the primary symptoms of stuttering. This hypothesis overlaps with ideas proposed by Zimmermann, Smith, and Hanley (1981), who suggested that stuttering moments can be interpreted in the context of interactions between a premovement “tuning of the reflex excitability” and movement-initiating “triggering signals to these muscle groups” as “a disorder of coordination may occur when either the tuning or the triggering inputs are aberrant” (p. 26). We currently interpret the combined empirical findings from our own series of experiments reviewed in this article as implicating the “tuning” component in stuttering. Of course, our view does not rule out the possibility that deficiencies in this component may themselves have developed as a result of, or in parallel with, deficiencies in the “triggering” component (i.e., leading to imprecise internal forward models—see the introduction). For example, other research groups have reported that, prior to speech onset, brain motor regions of individuals who stutter may show atypical oscillatory activity (Mersov, Cheyne, Jobst, & De Nil, 2018; Mersov, Jobst, Cheyne, & De Nil, 2016) and atypical excitation patterns (Whillier et al., 2018).

Support for our current interpretation that PSAM likely reflects processes involved in a complex optimization of auditory cortical activity in preparation for feedback monitoring after speech onset comes from multiple independent sources. First, findings from our own series of experiments strongly argue against a simple interpretation whereby PSAM is considered to reflect a general reduction of auditory input. We already mentioned above that a general suppression of cortical auditory input immediately prior to speech onset would be hard to reconcile with this sensory modality's dominant role in feedback-driven speech motor learning (Feng et al., 2011). In addition, as compared with speakers who stutter, speakers who do not stutter showed more PSAM than speakers who stutter, but they also relied more (rather than less) on auditory feedback in an auditory–motor adaptation task (Daliri & Max, 2018). Thus, at least at the level of group-based data, our findings are inconsistent with the idea that PSAM suppresses auditory feedback. Second, others have shown in human work with magnetoencephalography that the auditory cortex shows a stronger response to very small (not consciously detected) pitch shifts in the auditory input when this input is the feedback signal of active vocalization as opposed to a played-back prior vocalization (Franken et al., 2018). This observation indicates that the auditory system's sensitivity for monitoring spectral aspects of the speech signal is increased during vocalization. Third, and perhaps most important in terms of understanding the underlying mechanisms, similar conclusions follow from direct neurophysiological recordings in animal models of vocalization (Eliades & Tsunada, 2018; Eliades & Wang, 2008, 2017).

Studying self-initiated vocalization in marmoset monkeys, Eliades and colleagues (Eliades & Tsunada, 2018; Eliades & Wang, 2008) demonstrated that the suppression of cortical auditory neurons during self-initiated vocalization in fact increases these neurons' sensitivity to changes in the auditory feedback of the vocalization. Specifically, a large number of auditory neurons that were suppressed during self-vocalization with typical feedback became strongly excited when the animal heard feedback that was pitch-shifted by two semitones. Thus, these neurons' sensitivity to error was actually enhanced, and the authors concluded that “as a population, suppressed neurons were more sensitive to auditory feedback during vocalization than excited neurons, suggesting that they may have a greater role in vocal self-monitoring” (Eliades & Wang, 2008, p. 1103). In a separate study, the same authors showed that almost all auditory cortical neurons that are excited during vocalization are also excited by passive playback of vocalizations (suggesting a general sensory-driven response), whereas only a small proportion of neurons that are suppressed during vocalization are also suppressed by passive playback of vocalizations (Eliades & Wang, 2017). In other words, the majority of auditory neurons suppressed during self-vocalization are excited by played-back vocalization, suggesting that, prior to active vocalization, the excitability/inhibition of these neurons is modulated by auditory forward modeling mechanisms. One possibility is that efference copy signals bias the receptive fields of auditory cortical neurons in order to optimize vocal feedback encoding (Eliades & Wang, 2017). The net result during vocalization then is an increased sensitivity to auditory error as demonstrated in marmosets by Eliades and Wang (2008, 2017) and in humans by Franken et al. (2018), and the level of activation of at least some auditory neuronal populations appears to represent an error signal representing the extent of mismatch between predicted and actual feedback (Houde & Nagarajan, 2011; Niziolek et al., 2013).

It should be acknowledged that the studies described here also have a few limitations that should be considered, and possibly addressed, in future work. One limitation is that our data are based on EEG recordings, and this does not allow us to directly determine the neural substrates underlying PSAM. Nevertheless, by combining information regarding the known neural sources of the evoked N1 component (Godey, Schwartz, De Graaf, Chauvel, & Liégeois-Chauvel, 2001; Näätänen & Picton, 1987; Zouridakis, Simos, & Papanicolaou, 1998), the neural circuitry involved in the speech network (Dick, Bernal, & Tremblay, 2014; Glasser & Rilling, 2008; Hickok, 2012a, 2012b; Tremblay & Dick, 2016), and contemporary theoretical models of speech production (Guenther, 2016; Hickok, 2012b, 2013; Houde & Nagarajan, 2011), one can speculate that PSAM depends strongly on the pathway connecting ventral premotor and motor regions to primary and secondary auditory cortices. Structural neuroimaging studies of stuttering indeed have shown atypical findings in these pathways (Chang, Garnett, Etchell, & Chow, 2018; Chang, Zhu, Choo, & Angstadt, 2015; Sommer, Koch, Paulus, Weiller, & Büchel, 2002; Watkins, Smith, Davis, & Howell, 2008). It may be feasible in future studies to examine the possible relationship between PSAM (measured by means of EEG) and the integrity of these implicated pathways (measured by means of structural neuroimaging).

Another aspect of our work that potentially could be considered a limitation is the blocking of trials within conditions. That is, the total set of trials (typically 90) for each condition was split up into blocks (typically three), and then the total set of blocks (typically nine) from all conditions was presented in counterbalanced order across participants. Our rationale for this design has been that it allows participants to unambiguously know at all times whether speech should be produced on a given trial. The alternative option of randomizing all individual trials would (a) increase cognitive processing demands as participants would need to make a “speak” versus “do-not-speak” decision on each trial, (b) contaminate the EEG data with this decision-related activity, and (c) add an additional confounding variable as trials from the different conditions would also differ in the presence or absence of a separate response cue. However, it cannot be ruled out that this blocked design may introduce differences in other variables (e.g., attentional demand) across the conditions.

In conclusion, we discovered in a series of experiments that typically fluent adult speakers already modulate auditory processing mechanisms prior to the initiation of speech movements, whereas this PSAM mechanism is very limited or absent in adults who stutter unless the speech task is completed with DAF. In our prior publications on the individual experiments completed to date, we have described how the between-group difference in PSAM is consistently associated with large effect sizes, and in our most recent study, PSAM differentiated 100% between participants who stutter and participants who do not stutter (Daliri & Max, 2018). Consequently, PSAM is a particularly powerful phenomenon with potentially great promise for elucidating the mechanisms underlying speech motor breakdowns in stuttering. In currently ongoing work, we have therefore started investigating whether differences in PSAM are already present at a young age close to the onset of stuttering. In addition, we are initiating studies investigating whether the between-group difference in adults is specific to motor-to-auditory interactions or whether it is also observable in pre-speech motor-to-somatosensory interactions. Finally, we are designing new experimental paradigms that will allow us to directly test the above formulated hypotheses that PSAM actually enhances auditory sensitivity to production errors and that a lack of PSAM may lead to inefficient monitoring mechanisms and breakdowns in speech fluency. We hope that, in the long term, the results from this line of work will offer not only deeper insights into the physiological basis of stuttering but also suggestions for novel treatment approaches such as noninvasive brain stimulation of neural pathways involved in premovement motor-to-sensory signaling.

## References

[bib1] Altenmüller E., Berger W., Prokop T., Trippel M., & Dietz V. (1995). Modulation of sural nerve somatosensory evoked potentials during stance and different phases of the step-cycle. Electroencephalography and Clinical Neurophysiology/Evoked Potentials, 96(6), 516–525. https://doi.org/10.1016/0013-4694(95)00093-E 7489673 10.1016/0013-4694(95)00093-e

[bib2] Bays P. M., Wolpert D. M., & Flanagan J. R. (2005). Perception of the consequences of self-action is temporally tuned and event driven. Current Biology, 15(12), 1125–1128. https://doi.org/10.1016/j.cub.2005.05.023 15964278 10.1016/j.cub.2005.05.023

[bib3] Blakemore S.-J., Wolpert D. M., & Frith C. D. (1998). Central cancellation of self-produced tickle sensation. Nature Neuroscience, 1(7), 635–640. https://doi.org/10.1038/2870 10196573 10.1038/2870

[bib4] Blakemore S.-J., Wolpert D. M., & Frith C. D. (1999). The cerebellum contributes to somatosensory cortical activity during self-produced tactile stimulation. NeuroImage, 10(4), 448–459. https://doi.org/10.1006/nimg.1999.0478 10493902 10.1006/nimg.1999.0478

[bib5] Blakemore S.-J., Wolpert D. M., & Frith C. D. (2000). Why can't you tickle yourself? NeuroReport, 11(11), R11–R16. https://doi.org/10.1097/00001756-200008030-00002 10943682 10.1097/00001756-200008030-00002

[bib6] Bloodstein O., Ratner N. B., & Bernstein-Ratner N. (2008). A handbook on stuttering (6th ed., Vol. 6). Clifton Park, NY: Delmar.

[bib7] Cai S., Beal D. S., Ghosh S. S., Guenther F. H., & Perkell J. S. (2014). Impaired timing adjustments in response to time-varying auditory perturbation during connected speech production in persons who stutter. Brain and Language, 129(1), 24–29. https://doi.org/10.1016/j.bandl.2014.01.002 24486601 10.1016/j.bandl.2014.01.002 PMC3947674

[bib8] Cai S., Beal D. S., Ghosh S. S., Tiede M. K., Guenther F. H., & Perkell J. S. (2012). Weak responses to auditory feedback perturbation during articulation in persons who stutter: Evidence for abnormal auditory–motor transformation. PLOS ONE, 7(7), e41830 https://doi.org/10.1371/journal.pone.0041830 22911857 10.1371/journal.pone.0041830 PMC3402433

[bib9] Chang E. F., Niziolek C. A., Knight R. T., Nagarajan S. S., & Houde J. F. (2013). Human cortical sensorimotor network underlying feedback control of vocal pitch. Proceedings of the National Academy of Sciences of the United States of America, 110(7), 2653–2658. https://doi.org/10.1073/pnas.1216827110 23345447 10.1073/pnas.1216827110 PMC3574939

[bib10] Chang S.-E., Garnett E. O., Etchell A., & Chow H. M. (2018). Functional and neuroanatomical bases of developmental stuttering: Current insights. The Neuroscientist. Advance online publication. https://doi.org/10.1177/1073858418803594 10.1177/1073858418803594 PMC6486457 30264661

[bib11] Chang S.-E., Zhu D. C., Choo A. L., & Angstadt M. (2015). White matter neuroanatomical differences in young children who stutter. Brain, 138(3), 694–711. https://doi.org/10.1093/brain/awu400 25619509 10.1093/brain/awu400 PMC4339778

[bib12] Chapman C. E., Jiang W., & Lamarre Y. (1988). Modulation of lemniscal input during conditioned arm movements in the monkey. Experimental Brain Research, 72(2), 316–334. https://doi.org/10.1007/BF00250254 3224647 10.1007/BF00250254

[bib13] Civier O., Bullock D., Max L., & Guenther F. H. (2013). Computational modeling of stuttering caused by impairments in a basal ganglia thalamo-cortical circuit involved in syllable selection and initiation. Brain and Language, 126(3), 263–278. https://doi.org/10.1016/j.bandl.2013.05.016 23872286 10.1016/j.bandl.2013.05.016 PMC3775364

[bib14] Civier O., Tasko S. M., & Guenther F. H. (2010). Overreliance on auditory feedback may lead to sound/syllable repetitions: Simulations of stuttering and fluency-inducing conditions with a neural model of speech production. Journal of Fluency Disorders, 35(3), 246–279. https://doi.org/10.1016/j.jfludis.2010.05.002 20831971 10.1016/j.jfludis.2010.05.002 PMC2939043

[bib15] Cohen L. G., & Starr A. (1985). Vibration and muscle contraction affect somatosensory evoked potentials. Neurology, 35(5), 691–698. 3157885 10.1212/wnl.35.5.691

[bib16] Daliri A., & Max L. (2015a). Electrophysiological evidence for a general auditory prediction deficit in adults who stutter. Brain and Language, 150, 37–44. https://doi.org/10.1016/j.bandl.2015.08.008 26335995 10.1016/j.bandl.2015.08.008 PMC4663101

[bib17] Daliri A., & Max L. (2015b). Modulation of auditory processing during speech movement planning is limited in adults who stutter. Brain and Language, 143, 59–68. https://doi.org/10.1016/j.bandl.2015.03.002 25796060 10.1016/j.bandl.2015.03.002 PMC4380808

[bib18] Daliri A., & Max L. (2016). Modulation of auditory responses to speech vs. nonspeech stimuli during speech movement planning. Frontiers in Human Neuroscience, 10, 234 https://doi.org/10.3389/fnhum.2016.00234 27242494 10.3389/fnhum.2016.00234 PMC4870268

[bib19] Daliri A., & Max L. (2018). Stuttering adults' lack of pre-speech auditory modulation normalizes when speaking with delayed auditory feedback. Cortex, 99, 55–68. https://doi.org/10.1016/j.cortex.2017.10.019 29169049 10.1016/j.cortex.2017.10.019 PMC5801108

[bib20] Daliri A., Prokopenko R. A., Flanagan J. R., & Max L. (2014). Control and prediction components of movement planning in stuttering versus nonstuttering adults. Journal of Speech, Language, and Hearing Research, 57(6), 2131–2141. https://doi.org/10.1044/2014_JSLHR-S-13-0333 10.1044/2014_JSLHR-S-13-0333 PMC4270877 25203459

[bib21] Daliri A., Prokopenko R. A., & Max L. (2013). Afferent and efferent aspects of mandibular sensorimotor control in adults who stutter. Journal of Speech, Language, and Hearing Research, 56(6), 1774–1778. https://doi.org/10.1044/1092-4388(2013/12-0134) 10.1044/1092-4388(2013/12-0134) PMC3795963 23816664

[bib22] Daliri A., Wieland E. A., Cai S., Guenther F. H., & Chang S.-E. (2017). Auditory–motor adaptation is reduced in adults who stutter but not in children who stutter. Developmental Science, 21(2), e12521 https://doi.org/10.1111/desc.12521 10.1111/desc.12521 PMC5581739 28256029

[bib23] Dick A. S., Bernal B., & Tremblay P. (2014). The language connectome: New pathways, new concepts. Neuroscientist, 20(5), 453–467. https://doi.org/10.1177/1073858413513502 24342910 10.1177/1073858413513502

[bib24] Eliades S. J., & Tsunada J. (2018). Auditory cortical activity drives feedback-dependent vocal control in marmosets. Nature Communications, 9(1), 2540 https://doi.org/10.1038/s41467-018-04961-8 10.1038/s41467-018-04961-8 PMC6026141 29959315

[bib25] Eliades S. J., & Wang X. (2002). Sensory–motor interaction in the primate auditory cortex during self-initiated vocalizations. Journal of Neurophysiology, 89(4), 2194–2207. https://doi.org/10.1152/jn.00627.2002 12612021 10.1152/jn.00627.2002

[bib26] Eliades S. J., & Wang X. (2008). Neural substrates of vocalization feedback monitoring in primate auditory cortex. Nature, 453(7198), 1102–1106. https://doi.org/10.1038/nature06910 18454135 10.1038/nature06910

[bib27] Eliades S. J., & Wang X. (2017). Contributions of sensory tuning to auditory–vocal interactions in marmoset auditory cortex. Hearing Research, 348, 98–111. https://doi.org/10.1016/j.heares.2017.03.001 28284736 10.1016/j.heares.2017.03.001 PMC5392437

[bib28] Feng Y., Gracco V. L., & Max L. (2011). Integration of auditory and somatosensory error signals in the neural control of speech movements. Journal of Neurophysiology, 106(2), 667–679. https://doi.org/10.1152/jn.00638.2010 21562187 10.1152/jn.00638.2010 PMC3154803

[bib29] Franken M. K., Eisner F., Acheson D. J., McQueen J. M., Hagoort P., & Schoffelen J. M. (2018). Self-monitoring in the cerebral cortex: Neural responses to small pitch shifts in auditory feedback during speech production. NeuroImage, 179, 326–336. https://doi.org/10.1016/j.neuroimage.2018.06.061 29936308 10.1016/j.neuroimage.2018.06.061

[bib30] Glasser M. F., & Rilling J. K. (2008). DTI tractography of the human brain's language pathways. Cerebral Cortex, 18(11), 2471–2482. https://doi.org/10.1093/cercor/bhn011 18281301 10.1093/cercor/bhn011

[bib31] Godey B., Schwartz D., De Graaf J. B., Chauvel P., & Liégeois-Chauvel C. (2001). Neuromagnetic source localization of auditory evoked fields and intracerebral evoked potentials: A comparison of data in the same patients. Clinical Neurophysiology, 112(10), 1850–1859. https://doi.org/10.1016/S1388-2457(01)00636-8 11595143 10.1016/s1388-2457(01)00636-8

[bib32] Guenther F. H. (2016). Neural control of speech. Cambridge, MA: MIT Press.

[bib33] Heinks-Maldonado T. H., Mathalon D. H., Gray M., & Ford J. M. (2005). Fine-tuning of auditory cortex during speech production. Psychophysiology, 42(2), 180–190. https://doi.org/10.1111/j.1469-8986.2005.00272.x 15787855 10.1111/j.1469-8986.2005.00272.x

[bib34] Heinks-Maldonado T. H., Nagarajan S. S., & Houde J. F. (2006). Magnetoencephalographic evidence for a precise forward model in speech production. NeuroReport, 17(13), 1375–1379. https://doi.org/10.1097/01.wnr.0000233102.43526.e9 16932142 10.1097/01.wnr.0000233102.43526.e9 PMC4060597

[bib35] Hickok G. (2012a). Computational neuroanatomy of speech production. Nature Reviews Neuroscience, 13(2), 135–145. https://doi.org/10.1038/nrn3158 22218206 10.1038/nrn3158 PMC5367153

[bib36] Hickok G. (2012b). The cortical organization of speech processing: Feedback control and predictive coding the context of a dual-stream model. Journal of Communication Disorders, 45(6), 393–402. https://doi.org/10.1016/j.jcomdis.2012.06.004 22766458 10.1016/j.jcomdis.2012.06.004 PMC3468690

[bib37] Hickok G. (2013). The functional neuroanatomy of language. Physics of Life Reviews, 6(3), 121–143. https://doi.org/10.1016/j.plrev.2009.06.001 10.1016/j.plrev.2009.06.001 PMC2747108 20161054

[bib38] Houde J. F., & Chang E. F. (2015). The cortical computations underlying feedback control in vocal production. Current Opinion in Neurobiology, 33, 174–181. https://doi.org/10.1016/j.conb.2015.04.006 25989242 10.1016/j.conb.2015.04.006 PMC4628828

[bib39] Houde J. F., & Nagarajan S. S. (2011). Speech production as state feedback control. Frontiers in Human Neuroscience, 5, 82 https://doi.org/10.3389/fnhum.2011.00082 22046152 10.3389/fnhum.2011.00082 PMC3200525

[bib40] Houde J. F., Nagarajan S. S., Sekihara K., & Merzenich M. M. (2002). Modulation of the auditory cortex during speech: An MEG study. Journal of Cognitive Neuroscience, 14(8), 1125–1138. https://doi.org/10.1162/089892902760807140 12495520 10.1162/089892902760807140

[bib41] Kawato M., Kuroda T., Imamizu H., Nakano E., Miyauchi S., Yoshioka T., … Yoshioka T. (2003). Internal forward models in the cerebellum: fMRI study on grip force and load force coupling. Progress in Brain Research, 142, 171–188. https://doi.org/10.1016/S0079-6123(03)42013-X 12693261 10.1016/S0079-6123(03)42013-X

[bib42] Kim K. S., & Max L. (2014). Estimating feedforward vs. feedback control of speech production through kinematic analyses of unperturbed articulatory movements. Frontiers in Human Neuroscience, 8, 911 https://doi.org/10.3389/fnhum.2014.00911 25426056 10.3389/fnhum.2014.00911 PMC4227515

[bib43] Luck S. J. (2014). An introduction to the event-related potential technique (2nd ed.). Cambridge, MA: MIT Press.

[bib44] Max L. (2004). Stuttering and internal models for sensorimotor control: A theoretical perspective to generate testable hypotheses. In Maassen B., Kent R., Peters H. F. M., van Lieshout P., & Hulstijn W. (Eds.), Speech motor control in normal and disordered speech (pp. 357–387). Oxford, United Kingdom: Oxford University Press.

[bib45] Max L., Daniels J. C., Curet K. M., & Cronin K. L. (2008). Modulation of auditory and somatosensory processing during the planning of speech movements. Proceedings of the 8th International Seminar on Speech Production, 2, 41–44.

[bib46] Max L., Guenther F. H., Gracco V. L., Ghosh S. S., & Wallace M. E. (2004). Unstable or insufficiently activated internal models and feedback-biased motor control as sources of dysfluency: A theoretical model of stuttering. Contemporary Issues in Communication Science and Disorders, 31, 105–122.

[bib47] Merrikhi Y., Ebrahimpour R., & Daliri A. (2018). Perceptual manifestations of auditory modulation during speech planning. Experimental Brain Research, 236(7), 1963–1969. https://doi.org/10.1007/s00221-018-5278-3 29713756 10.1007/s00221-018-5278-3

[bib48] Mersov A.-M., Cheyne D. O., Jobst C., & De Nil L. F. (2018). A preliminary study on the neural oscillatory characteristics of motor preparation prior to dysfluent and fluent utterances in adults who stutter. Journal of Fluency Disorders, 55, 145–155. https://doi.org/10.1016/j.jfludis.2017.05.003 28577876 10.1016/j.jfludis.2017.05.003

[bib49] Mersov A.-M., Jobst C., Cheyne D. O., & De Nil L. F. (2016). Sensorimotor oscillations prior to speech onset reflect altered motor networks in adults who stutter. Frontiers in Human Neuroscience, 10, 443 https://doi.org/10.3389/fnhum.2016.00443 27642279 10.3389/fnhum.2016.00443 PMC5009120

[bib50] Mock J. R., Foundas A. L., & Golob E. J. (2015). Speech preparation in adults with persistent developmental stuttering. Brain and Language, 149, 97–105. https://doi.org/10.1016/j.bandl.2015.05.009 26197258 10.1016/j.bandl.2015.05.009 PMC4586364

[bib51] Näätänen R., & Picton T. (1987). The N1 wave of the human electric and magnetic response to sound: A review and an analysis of the component structure. Psychophysiology, 24(4), 375–425. 3615753 10.1111/j.1469-8986.1987.tb00311.x

[bib52] Namasivayam A. K., van Lieshout P. H. M., McIlroy W. E., & De Nil L. F. (2009). Sensory feedback dependence hypothesis in persons who stutter. Human Movement Science, 28(6), 688–707. https://doi.org/10.1016/j.humov.2009.04.004 19692132 10.1016/j.humov.2009.04.004

[bib53] Niziolek C. A., Nagarajan S. S., & Houde J. F. (2013). What does motor efference copy represent? Evidence from speech production. Journal of Neuroscience, 33(41), 16110–16116. https://doi.org/10.1523/JNEUROSCI.2137-13.2013 24107944 10.1523/JNEUROSCI.2137-13.2013 PMC3792453

[bib54] Numminen J., Salmelin R., & Hari R. (1999). Subject's own speech reduces reactivity of the human auditory cortex. Neuroscience Letters, 265(2), 119–122. https://doi.org/10.1016/S0304-3940(99)00218-9 10327183 10.1016/s0304-3940(99)00218-9

[bib55] Nunez P. L., & Srinivasan R. (2006). Electric fields of the brain: the neurophysics of EEG (2nd ed.). London, United Kingdom: Oxford University Press.

[bib56] Seki K., Perlmutter S. I., & Fetz E. E. (2003). Sensory input to primate spinal cord is presynaptically inhibited during voluntary movement. Nature Neuroscience, 6(12), 1309–1316. https://doi.org/10.1038/nn1154 14625555 10.1038/nn1154

[bib57] Shadmehr R., Smith M. A., & Krakauer J. W. (2010). Error correction, sensory prediction, and adaptation in motor control. Annual Review of Neuroscience, 33(1), 89–108. 10.1146/annurev-neuro-060909-153135 20367317

[bib58] Sommer M., Koch M. A., Paulus W., Weiller C., & Büchel C. (2002). Disconnection of speech-relevant brain areas in persistent developmental stuttering. Lancet, 360(9330), 380–383. https://doi.org/10.1016/S0140-6736(02)09610-1 12241779 10.1016/S0140-6736(02)09610-1

[bib59] Sowman P. F., Brinkworth R. S. A., & Türker K. S. (2010). Threshold for detection of incisal forces is increased by jaw movement. Journal of Dental Research, 89(4), 395–399. https://doi.org/10.1177/0022034510363101 20200410 10.1177/0022034510363101

[bib60] Tourville J. A., & Guenther F. H. (2011). The DIVA model: A neural theory of speech acquisition and production. Language and Cognitive Processes, 26(7), 952–981. https://doi.org/10.1080/01690960903498424 23667281 10.1080/01690960903498424 PMC3650855

[bib61] Tremblay P., & Dick A. S. (2016). Broca and Wernicke are dead, or moving past the classic model of language neurobiology. Brain and Language, 162, 60–71. https://doi.org/10.1016/j.bandl.2016.08.004 27584714 10.1016/j.bandl.2016.08.004

[bib62] Voss M., Ingram J. N., Haggard P., & Wolpert D. M. (2006). Sensorimotor attenuation by central motor command signals in the absence of movement. Nature Neuroscience, 9(1), 26–27. https://doi.org/10.1038/nn1592 16311591 10.1038/nn1592 PMC2636578

[bib63] Walsh E., & Haggard P. (2008). The effects of acoustic startle on sensorimotor attenuation prior to movement. Experimental Brain Research, 189(3), 279–288. https://doi.org/10.1007/s00221-008-1421-x 18493751 10.1007/s00221-008-1421-x

[bib64] Walsh E., & Haggard P. (2010). Somatosensory effects of action inhibition: A study with the stop-signal paradigm. Experimental Brain Research, 204(3), 465–473. https://doi.org/10.1007/s00221-010-2181-y 20165838 10.1007/s00221-010-2181-y

[bib65] Watkins K. E., Smith S. M., Davis S., & Howell P. (2008). Structural and functional abnormalities of the motor system in developmental stuttering. Brain, 131(1), 50–59. https://doi.org/10.1093/brain/awm241 17928317 10.1093/brain/awm241 PMC2492392

[bib66] Weiskrantz L., Elliott J., & Darlington C. (1971). Preliminary observations on tickling oneself. Nature, 230(5296), 598–599. https://doi.org/10.1038/230598a0 4928671 10.1038/230598a0

[bib67] Whillier A., Hommel S., Neef N. E., Von Gudenberg A. W., Paulus W., & Sommer M. (2018). Adults who stutter lack the specialised pre-speech facilitation found in non-stutterers. PLOS ONE, 13(10), 1–27. https://doi.org/10.1371/journal.pone.0202634 10.1371/journal.pone.0202634 PMC6179203 30303960

[bib68] Williams S. R., & Chapman C. E. (2002). Time course and magnitude of movement-related gating of tactile detection in humans. III. Effect of motor tasks. Journal of Neurophysiology, 88(4), 1968–1979. https://doi.org/10.1152/jn.00527.2001 12364522 10.1152/jn.2002.88.4.1968

[bib69] Wolpert D. M., Diedrichsen J., & Flanagan J. R. (2011). Principles of sensorimotor learning. Nature Reviews Neuroscience, 12(12), 739–751. https://doi.org/10.1038/nrn3112 22033537 10.1038/nrn3112

[bib70] Wolpert D. M., & Flanagan J. R. (2001). Motor prediction. Current Biology, 11(18), R729–R732. https://doi.org/10.1016/S0960-9822(01)00432-8 11566114 10.1016/s0960-9822(01)00432-8

[bib71] Wolpert D. M., Miall R. C., & Kawato M. (1998). Internal models in the cerebellum. Trends in Cognitive Sciences, 2(9), 338–347. https://doi.org/10.1016/S1364-6613(98)01221-2 21227230 10.1016/s1364-6613(98)01221-2

[bib72] Yang S. C. H., Wolpert D. M., & Lengyel M. (2016). Theoretical perspectives on active sensing. Current Opinion in Behavioral Sciences, 11, 100–108. https://doi.org/10.1016/j.cobeha.2016.06.009 10.1016/j.cobeha.2016.06.009 PMC6116896 30175197

[bib73] Zimmermann G. N., Smith A., & Hanley J. M. (1981). Stuttering: in need of a unifying conceptual framework. Journal of Speech and Hearing Research, 24(1), 25–31. 7253624

[bib74] Zouridakis G., Simos P. G., & Papanicolaou A. C. (1998). Multiple bilaterally asymmetric cortical sources account for the auditory N1m component. Brain Topography, 10(3), 183–189. https://doi.org/10.1023/A:1022246825461 9562539 10.1023/a:1022246825461

